# Letter in response to *Relationship between primary eradication of Helicobacter pylori and drinking habits in women: collaborative research between a pharmacy and a clinic*

**DOI:** 10.1017/S095026881900219X

**Published:** 2020-01-10

**Authors:** D. W. Mayderry

**Affiliations:** Department of Epidemiology, University of Florida, 2004 Mowry Road, PO Box 100231, Gainesville, FL 32610, USA

To the Editor

The recently published work, ‘Relationship between primary eradication of *Helicobacter pylori* and drinking habits in women: collaborative research between a pharmacy and clinic’ by Ozeki *et al*. is of great interest to researchers and students studying gut dysbiosis. Ozeki *et al*. conclude that the rates of *H. pylori* eradication are lower in women who drink between 5 and 7 days out of the week, and among other factors, this was likely due to differing abilities of men and women to metabolise proton pump inhibitors in the presence of alcohol [1].

Recognition is due to the authors for the timely study of a common but life altering infection, especially considering the recent rise in eradication failure of *H. pylori* in Japan [2]. In addition, linking pharmacists, who are in an exceptional position to play a role in community health research, is an excellent and resourceful use of existing structures to promote health and wellness in community settings. Despite these facts, four additional limitations to study design that were not mentioned could have played a role in the outcomes noted in the results and discussion section of this article.

First, data on the consumption of probiotics supplements, or probiotic-rich foods such as yogurt and kimchi, were not gathered. Fermented foods and probiotics, specifically those containing lactic acid-producing bacteria, are known to suppress *H. pylori* growth and improve eradication rates [3, 4, 5]. Second, data regarding past *H. pylori* infection were not gathered from participants. Past infection with *H. pylori* is known to strongly increase the risk of antimicrobial resistance and subsequent eradication failure, especially in the case of clarithromycin [6]. The most common cause of *H. pylori* eradication failure is resistance to frequently prescribed antibiotics, including clarithromycin [6]. Third, participants of this study were not asked about antibiotic use or adherence to past antibiotic regimens, which could further predict antimicrobial resistance and limit success of eradication [6]. Fourth, those who drink have been shown to be less likely to take prescribed medication as required, furthering adherence-related eradication failure [7].

The findings of this study are novel, and point to underlying mechanisms in *H. pylori* infection that require further investigation into potential infection disparities between men and women who use alcohol during eradication therapy.
